# Obesity, Sodium Homeostasis, and Arterial Hypertension in Children and Adolescents

**DOI:** 10.3390/nu13114032

**Published:** 2021-11-11

**Authors:** Małgorzata Wójcik, Agnieszka Kozioł-Kozakowska

**Affiliations:** 1 Department of Pediatric and Adolescent Endocrinology, Chair of Pediatrics, Pediatric Institute, Jagiellonian University Medical College, 30-663 Kraków, Poland; 2 Department of Pediatrics, Gastroenterology and Nutrition, Institute of Pediatrics, Jagiellonian University Medical College, 30-663 Kraków, Poland; agnieszka.koziol-kozakowska@uj.edu.pl

**Keywords:** obesity, hypertension, salt, sodium, children

## Abstract

Background: The relationship between obesity, arterial hypertension, and excessive salt intake has been known for a long time; however, the mechanism of this relationship remains not clear. Methods: The paper presents a current literature review on the relationship between salt consumption and the development of arterial hypertension in children and adolescents with obesity. Results: In addition to the traditional theory of hypertension development due to the increase in intravascular volume and disturbances of sodium excretion, recent studies indicate the existence of a complex mechanism related to excessive, pathological secretory activity of adipocytes, insulin resistance, and impaired function of the renin–angiotensin–aldosterone axis. That makes obese children and adolescents particularly vulnerable to the development of salt-sensitive arterial hypertension. Studies performed in many countries have shown that children and adolescents consume more sodium than recommended. It is worth noting, however, that the basis for these recommendations was the extrapolation of data from studies conducted on adults. Moreover, more important than sodium intake is the Na/K ratio and water consumption. Conclusion: Regardless of the population-wide recommendations on reducing salt intake in children, specific recommendations for overweight and obese patients should be developed.

## 1. Introduction

According to the latest epidemiological studies, the prevalence of childhood arterial hypertension in 2015 ranged from 4.32% among children aged 6 years to 3.28% among those aged 9 years and peaked at 7.89% among those aged 14 years [1]. Currently, the frequency is even more difficult to define because there are differences regarding the threshold values for different pediatric age groups in the European and American guidelines and some local, national pediatric reference values [2,3]. In addition, the data regarding the incidence of arterial hypertension may be influenced by the lowering of the thresholds due to the exclusion of those who are obese and overweight from datasets and the desire to align thresholds for older and taller teens with adult thresholds. At the same time, 6% of girls and 8% of boys were suffering from obesity [4]. The situation becomes even more complex if you notice that arterial hypertension occurs in 10 to approximately 30% of children with obesity, while only <5% in the normal-weight pediatric population [5,6]. Some studies pointed to a link between obesity, hypertension, and excessive salt intake. It has been shown that daily salt intake in children is correlated with the prevalence of obesity and arterial hypertension [7]. The importance of salt consumption for the development of arterial hypertension has been known for over a hundred years [8]. The relationship between obesity and arterial hypertension development was first described half a century later [9]. However, the details of these relationships remain unclear to date. It remains unclear which factors determine the ‘salt-sensitivity’ phenomenon in the development of arterial hypertension. It remains unclear which factors contribute to the ‘salt-sensitivity’ phenomenon in the development of obesity-related hypertension. Recently, the evidence is growing for the contribution of excessive fat tissue and its biological activity, as will be reviewed here.

‘Salt’, properly sodium chloride (NaCl), is the main form of sodium intake. Salt is an essential ingredient in food processing. It is also one of the most widely used flavor enhancers. Additional sources of sodium are other substances added during the production of processed foods: sodium bicarbonate in fine bakery wares or sodium nitrate in processed meat [10]. It is estimated that the amount of salt delivered with food in the modern Western diet significantly exceeds the body’s actual needs. Many studies have shown that sodium intake in children is between 100% and 250% higher than the recommended value [11,12,13,14,15,16]. However, it should be noticed, that the recommendations for children’s salt intake are based on extrapolation of the data from adult studies. Moreover, no differentiation has been made between recommendations for healthy children and those for children with obesity and/or hypertension to date. The discoveries of recent years, which have made possible better understanding of the mechanisms linking salt intake, obesity, and hypertension, indicate the need for greater individualization of recommendations. The present paper is a review of the literature regarding the relationship between salt consumption, obesity, and the development of arterial hypertension in children.

## 2. Recommendations vs. Real Salt Intake in a Pediatric Population

The basis of and the crucial element in the treatment of children with obesity and arterial hypertension is the reduction of the fat tissue. First, it is recommended to change eating habits and increase physical activity. The diet should be focused on appropriate energy balance and a DASH (the Dietary Approaches to Stop Hypertension diet)-like pattern [17]. The DASH diet pattern is rich in fruit and vegetables, low-fat or fat-free dairy products, whole grains, fish, poultry, beans, seeds, and nuts, and lower in sweets and added sugars, fats, and red meat than the typical Western diet. The DASH diet substantially reduces both systolic and diastolic BPs among adults with stage 1 hypertension or prehypertension [18] and adolescents with stage 1 hypertension [19]. The World Health Organization (WHO) recommends a reduction in sodium intake for better control of blood pressure in children aged 2–15 years. The recommended maximum daily intake of 2000 mg in adults should be adjusted downward based on the energy requirements of children relative to those of adults [20]. The European Food Safety Authority (EFSA) Panel on Nutrition states that sodium intakes considered safe and adequate for children are extrapolated from the value for adults, adjusting for their respective energy requirement and growth: 1100 mg/day for children aged 1–3 years, 1300 mg/day for children aged 4–6 years, 1700 mg/day for children aged 7–10 years, and 2000 mg/day for children aged 11–17 years, respectively. For infants aged 7–11 months, an adequate intake (AI) of 200 mg/day is proposed based on upwards extrapolation of the estimated sodium intake in exclusively breast-fed infants aged 0–6 months [10]. Unfortunately, recommendations for salt restriction are not uniform (e.g., EFSA, WHO - 2000 mg, US AGR Dep - 2300 mg); there is no hard evidence for whether these extrapolated values are optimal for children and adolescents and, furthermore, we do not have specific, validated recommendations for pediatric patients with obesity and/or arterial hypertension. Nevertheless, it is certain that the population consumption of sodium significantly differs from the recommendation. The median sodium intake for individuals aged 4–17 years (*n* = 16,013) between 2003 and 2016 assessed in the US National Health and Nutrition Examination Surveys (NHANES) was 2840 mg/day (95% CI, 2805–2875 mg/day), decreasing from 2912 mg/day (95% CI 2848–2961 mg/day) in 2003–2004 to 2787 mg/day (95% CI, 2677–2867 mg/day) in 2015–2016. The mean intake increased with age: 2507 mg/day for individuals aged 4–8 years, 2934 mg/day for those aged 9–13 years, and 3124 mg/day for those aged 14–17 years; and was greater in males than in females (3053 mg/day vs. 2624 mg/day) [21]. Similar results were obtained in the Polish study that revealed an average excess of recommended sodium intake in preschool children of 82% [22]. Likewise, in Portuguese adolescents, 83% had a sodium intake above the upper limit [23]. In French adolescents, median sodium intake was 2245 mg/day [24]. In Australian adolescents (aged 14–16 years), mean dietary sodium intake was even higher, 3190 mg/day, and that value increased with age [25]. Similar results were also obtained using a more objective method than diary analysis—assessment of 24-h urinary sodium excretion. Mean sodium excretion in adolescents was: 3072 mg/day in Portugal, 2967 mg/day in Italy, 3270.6 mg/day in Spain, 3401.47 mg/day in England, and 3013 mg/day in Germany [23,26,27,28,29].

## 3. Salt Consumption and a Risk of Arterial Hypertension in Children with Obesity

The relationship between excessive salt consumption, increase in body weight, and elevated blood pressure has been supported by numerous studies [30,31,32,33,34]. It has been known for years that excessive salt consumption is correlated with higher incidence of arterial hypertension at the population level, however, the mechanism of this relationship remains not obvious. The association of obesity and salt sensitivity was explored by Roccini et al. in 1989. They revealed that the blood pressure of obese adolescents is sensitive to dietary sodium intake and that this sensitivity may be due to the combined effects of hyperinsulinemia, hyperaldosteronism, and increased activity of the sympathetic nervous system: all characteristic for obesity [35]. A recently published literature review including 6572 publications and 85 studies with 58,531 participants has confirmed that sodium intake is positively associated with blood pressure value in children and adolescents, with consistent findings in experimental and observational studies [36]. A Portuguese cross-sectional study revealed, that higher salt intake was associated with higher systolic blood pressure in boys aged 8–9 years, especially in those who were overweight or obese [37]. In one study, it was calculated that the risk for prehypertension increased by 35% for each additional gram of sodium per day among normal-weight participants and by 74% among overweight and obese children [38]. It has been shown that weight loss improved salt sensitivity in adolescents [35]. Additionally, salt sensitivity increases with the occurrence of complications typical of the metabolic syndrome [39]. These literature data provide strong evidence for an association between excessive salt intake, obesity, and hypertension in a pediatric population.

## 4. Proposed Mechanisms of Sodium Induced Hypertension in Obesity

### 4.1. Increased Extracellular Fluid Volume and Impaired Sodium Excretion

According to the traditional model of the relationship between salt consumption and the development of arterial hypertension in obese individuals, it is associated with increased extracellular fluid volume and higher blood flow in numerous tissues, also in the kidney [40,41]. Increased renal blood flow and accelerated glomerular filtration rate enhance renal sodium reabsorption. Subsequently, blood pressure increases, leading to further glomerular hyperfiltration with concomitant neurohumoral activation, leading to glomerular injury, impaired renal sodium excretion capacity and, finally, to the gradual loss of nephron function. This theory pointed to the disturbance of sodium excretion as the main factor leading to kidney damage and the development of arterial hypertension in obese individuals [42]. This classical theory, which is certainly the core of the pathogenesis of arterial hypertension in obesity, seems to be incomplete, however, and has been revised recently [42]. Some recently published studies indicate the existence of many complex mechanisms leading to the development of salt-sensitive hypertension in obese individuals. According to the classical theory of salt-sensitive arterial hypertension development, a strict salt restriction should be of universal benefit.

### 4.2. Mineralocorticoids/Mineralocorticoid Receptor

The discoveries of recent years allow for a better understanding of the pathogenesis of hypertension in obese people and indicate the special role of salt consumption in this process. The key element seems to be the previously underestimated role of mineralocorticoids/the mineralocorticoid receptor and their synergistic relationship with salt excess [43,44]. Excessive activity of the renin–angiotensin–aldosterone system is known to increase blood pressure. In healthy, normotensive, non-obese individuals, renin is released from the kidneys as a reaction to a decrease of blood volume or sodium concentration level. Increased renin levels generate angiotensin I synthesis, which subsequently stimulates vasoconstriction—as a consequence, blood pressure increases. Angiotensin II stimulates the adrenal cortex to secrete aldosterone, which additionally maintains the correct volume through sodium retention. On the contrary, if blood pressure rises or salt intake is excessive, the kidneys reduce the release of renin, which allows them to excrete more sodium and restores normal blood pressure. Angiotensin II is responsible for short-term, immediate pressure regulation, while the sodium-volume mechanism provides long-term control [45]. However, in obese individuals, that classic way of activating the renin–angiotensin–aldosterone system is affected by disturbing processes. Contrary to lean hypertensive subjects, patients with obesity show a positive paradoxical correlation between sodium intake and aldosterone levels. In non-obese patients, excessive sodium intake is expected to decrease aldosterone secretion by inhibiting the renin–angiotensin system [46]. This effect is greatly diminished in patients with obesity. This lack of a salt inhibitory effect on the activity of the renin–angiotensin–aldosterone axis in obese subjects appears to be caused by several adipose tissue-derived factors. Based on animal studies, it has been suggested that some adipokines, as yet unidentified, may directly stimulate aldosterone release from adrenals in a way independent from angiotensin II [43,47]. Moreover, it has been proven that angiotensinogen, angiotensin I, and angiotensin II may be produced directly in adipocytes and may stimulate adipose tissue cells to induce local production of aldosterone by autocrine/paracrine stimulation independent from the inhibitory effect of excessive salt consumption [48]. Another possible factor is the decreased levels of adiponectin found in obese individuals. Adiponectin, one of the first described adipocytokines, has a significant role in the inhibition of renin secretion. Thus, its reduced concentration level leads to an increase in the activity of the renin–angiotensin–aldosterone axis, regardless of salt intake [49]. Additionally, animal and human studies suggest reduced adiponectin levels may also contribute to HTN by causing endothelial dysfunction [50]. Another element that should be taken into consideration in the analysis of the pathology of arterial hypertension in obese people is vitamin D deficiency, which is often found in that group of patients [51]. Vitamin D has an inhibitory effect on renin secretion. Vitamin D deficiency thus leads directly to an increase in renin levels, independent of salt intake [49]. Many observational studies confirmed association between vitamin D deficiency and a higher risk of hypertension [52,53,54,55]. However, there has been no beneficial effects of vitamin D supplementation on a reduction in arterial pressure [51]. One of the more recent observations is that, in humans, the combined intake of salt and glucose, so common in highly processed food or fast food, also negatively affects the regulation of the renin–angiotensin–aldosterone system, irrespective of body weight [56]. As the details of the responsible mechanism remain unclear, that observation needs to be confirmed in further studies.

**Figure 1:**
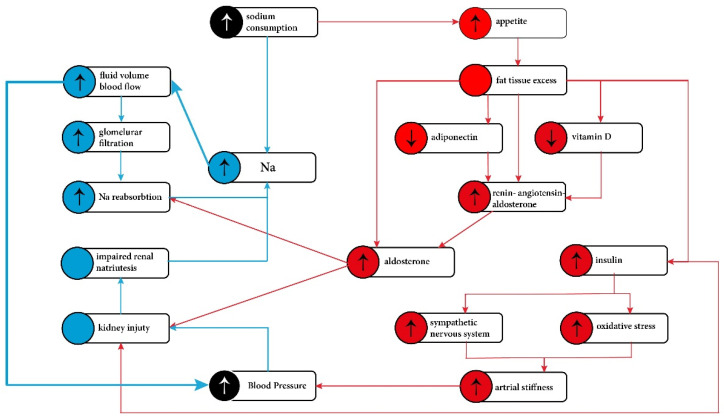
Pathophysiological mechanisms of the hypertension development in obese individuals. Blue—‘traditional pathway’. Red—new concepts involving fat tissue role. The traditional pathway was directed to the kidney and impaired sodium excretion. It is now known that the mechanism of the development of arterial hypertension in obesity is more complex and is associated with excess adipose tissue and abnormal adipocytes function.

All these newly discovered mechanisms described above shift weight from the simple mechanical volumetric overload of glomerular filtration towards more complex hormonal interplay with a key player: aldosterone. Research results of recent years have shown that aldosterone is not only the hormone that regulates electrolytes and fluid volume, but can be an important mediator of obesity development independent of calorie intake and target organ damage. An excess of aldosterone stimulates high sodium intake, induces insulin resistance, leptin resistance, adipose tissue inflammation, and impairs thermogenesis in brown adipose tissue and thus could contribute to the development of obesity and related metabolic abnormalities [48]. Moreover, high sodium intake causes an acceleration of fat tissue accumulation [57]. In addition, obese children also show enhanced production and abnormal circadian rhythm of cortisol [58,59]. Cortisol that is produced in the adipose tissue from inactive cortisone by 11β-hydroxysteroid dehydrogenase, may stimulate renin production, similarly to aldosterone [42]. That can exert aldosterone-like effects through its mineralocorticoid activity and, moreover, may increase insulin resistance [58]. Many of the processes associated with excess body fat and excessive salt intake that lead to the development of hypertension resemble a vicious circle or even a vicious spiral pattern (see Figure 1). In the kidney, mineralocorticoids cause podocyte injury, leading to proinflammatory response, mediating perivascular and interstitial fibrosis, glomerulosclerosis, and finally proteinuria [60].

### 4.3. Hyperinsulinemia/Insulin Resistance

Hyperinsulinemia and insulin resistance have a similar, additive effect leading to direct kidney damage. Insulin resistance, actually reduction in phosphoinositide 3-kinase/protein kinase B signaling, causes a reduction in nitric oxide synthesis that leads to the impairment of tubule-glomerular feedback, subsequent hyperfiltration, and sodium retention [61]. The small increase in plasma sodium also stimulates the thirst center, leading to water intake and secretion of arginine vasopressin, resulting in water retention [62]. These mechanisms restore plasma sodium to its previous level, but also increase extracellular fluid volume. That subsequently stimulates other compensatory mechanisms involved in the autoregulatory effect on resistance vessels. As a result, the blood pressure value increases. An important link between fat tissue and arterial hypertension development is increased activity of the sympathetic nervous system. The over-secretion of leptin and insulin are also implicated in stimulation of the sympathetic nervous system through the proopiomelanocortin-melanocortin 4 receptor pathway in the central nervous system [41,63]. As a consequence, peripheral vascular stiffness increases, kidney damage progresses, and sodium exertion is further impaired [64]. Additionally, insulin resistance and excessive pro-inflammatory activity of fat tissue are both responsible for oxidative stress development and endothelial damage [65].

## 5. Directions in the Development of Salt Intake Recommendations, including Individualization in Patients with Obesity

Recent data regarding sodium intake show that populations around the world consume significantly more sodium than their physiological needs [36,38,66,67]. As a result of this situation, the WHO has identified sodium restriction as a priority issue [68]. Interestingly, there are some studies that show that increased salt intake does not significantly affect blood pressure in people who do not suffer from hypertension. Based on the analysis of the data obtained from 6985 adults with no prior history of hypertension who participated in the National Health and Nutrition Examination Survey (2001–2006), no association between higher quartiles of sodium intake and the risk of a blood pressure value >140/90 mmHg or >130/80 mmHg was found [69]. Thus, it seems that not all individuals benefit equally from limiting dietary sodium [69]. It is also worth noting that not only sodium dose, but its ratio to potassium might be crucial. The relative potassium deficiency (in relation to the amount of consumed sodium) seems to be more important as the cause of hypertension than the absolute amount of sodium in the diet [70]. A high potassium intake and a low Na/K ratio appear to positively affect the physiological rise of blood pressure in childhood, resulting in smaller blood pressure slopes [71]. The potassium increases urinary sodium excretion which diminishes body sodium [72]. In addition, potassium is thought to decrease peripheral resistance [73]. Increased potassium intake can balance out excessive sodium consumption [74,75]. The WHO recommends a potassium intake of at least 3510 mg/day. However, a recently published meta-analysis identified a nonlinear relationship between potassium intake and blood pressure. An adequate intake of potassium is desirable to achieve a lower blood pressure, but excessive potassium supplementation should be avoided [76].

The diet rich in fruits and vegetables, low-fat dairy products, and low saturated and total fat—the so-called DASH diet mentioned before—helps to fulfill these recommendations as well as the normalization of body weight. The current European Society for Paediatric Gastroenterology Hepatology and Nutrition (ESPGHAN) recommendations emphasize that it is not a specific diet pattern (Mediterranean, vegetarian, vegan, or Nordic), but a complex of factors related to nutritional behavior, mainly that of parents, that are of key importance in the prevention of obesity [77]. Additionally, it has been shown that increased water consumption may have a positive effect on the reduction of blood pressure in children consuming comparable amounts of salt [78].

## 6. Conclusions

Now it is clear, that the relationship between salt consumption and arterial hypertension in pediatric patients with obesity is not obvious. The mechanical effect of renal compression and the factors increasing the activity of the sympathetic nervous system cannot be overlooked either. The recommendations for a universal reduction in salt intake may not be as sufficient as it was thought to be. It seems important to focus on the particular risk group of obese and/or hypertensive children. Especially for these children, the diet management should focus on reducing the amount of sodium while increasing the consumption of potassium, which, in practice, in most cases means an increase in the consumption of vegetables and fruits (fruits portion adjusted to the energy requirements).

## Data Availability

Not applicable.
